# Pseudobulbar Affect Presenting as Aggressive Behavior

**DOI:** 10.7759/cureus.21978

**Published:** 2022-02-07

**Authors:** Sana Elham Kazi, Adeel Anwar

**Affiliations:** 1 Psychiatry, Brookdale University Hospital Medical Center, Brooklyn, USA

**Keywords:** cannabis use, dextromethorphan-quinidine, aphasia, stroke, aggressive behavior, pseudobular afffect

## Abstract

Pseudobulbar affect (PBA) is an affective disorder of emotional expression characterized by frequent uncontrollable outbursts of laughing or crying. It is usually associated with stroke, traumatic brain injury, and other neurological conditions. This disorder can present a challenge to clinicians to distinguish this from mood disorders or to diagnose this disorder in the context of underlying mood disorders. In addition, the delay in the diagnosis can impact patients' quality of life.

We describe a 48-year-old man who presented with frequent episodes of sudden, frequent, uncontrollable laughing two years after his recurrent stroke. The patient initially had his first stroke about three years ago and had a recurrent stroke eight months after his first stroke. A few days after getting discharged after his second stroke, the patient was admitted to the psychiatric unit after his family members reported aggressive behavior. The patient also reported symptoms of depression and was discharged on escitalopram for mood and divalproex for his aggressive behavior. Unfortunately, the patient was not compliant with these medications with no resolution of his symptoms. The patient was then treated with dextromethorphan-quinidine, escitalopram, and divalproex, resulting in significant improvement in his mood and aggressive behavior with a resolution of uncontrollable laughing spells.

Clinicians are encouraged to inquire about symptoms of pseudobulbar affect in the context of stroke or other neurological disorders. Appropriate management of this condition can help improve patients' symptoms and positively affect their wellbeing.

## Introduction

Pseudobulbar affect (PBA) is an affective disorder of emotional expression. It is associated with stroke, traumatic brain injury, brain tumor, Alzheimer's dementia, Parkinson's disease, multiple sclerosis, and motor neuron disorders like amyotrophic lateral sclerosis [1-3]. 

PBA is characterized by sudden involuntary emotional expression with labile affect and episodes of uncontrollable, inappropriate, exaggerated laughing or crying. This disorder can present a challenge to clinicians to distinguish this from mood disorders and can be misdiagnosed as major depressive disorder, persistent depressive disorder, or bipolar disorder [4]. The difference between mood disorders and PBA is the duration. Depression symptoms, including depressed mood, typically last weeks to months and years, while PBA episodes last seconds to minutes. In addition, crying, as a symptom of PBA, may be unrelated to mood, while crying is consistent with the emotional state in depression. Other symptoms of depression, such as fatigue, anorexia, insomnia, anhedonia, and feelings of hopelessness and guilt, are not associated with PBA. Similarly, PBA can be differentiated from bipolar disorders based on the relatively brief duration of laughing or crying episodes (with no mood disturbance between episodes), compared with the sustained changes in mood, cognition, and behavior noted in bipolar disorders [5].

PBA is often recognized late in the disease, contributing to patients' poor quality of life. Therefore, PBA should be strongly considered in patients with stroke. About 20% of stroke survivors demonstrate acute PBA within six months of stroke, and about 12% show PBA symptoms after six months post-stroke [6].

We present a case of a patient with repeated cerebrovascular events who presented with confusion and aggressive and violent behaviors. He also had inappropriate laughter and crying spells suggestive of PBA. 

## Case presentation

**Figure 1:**
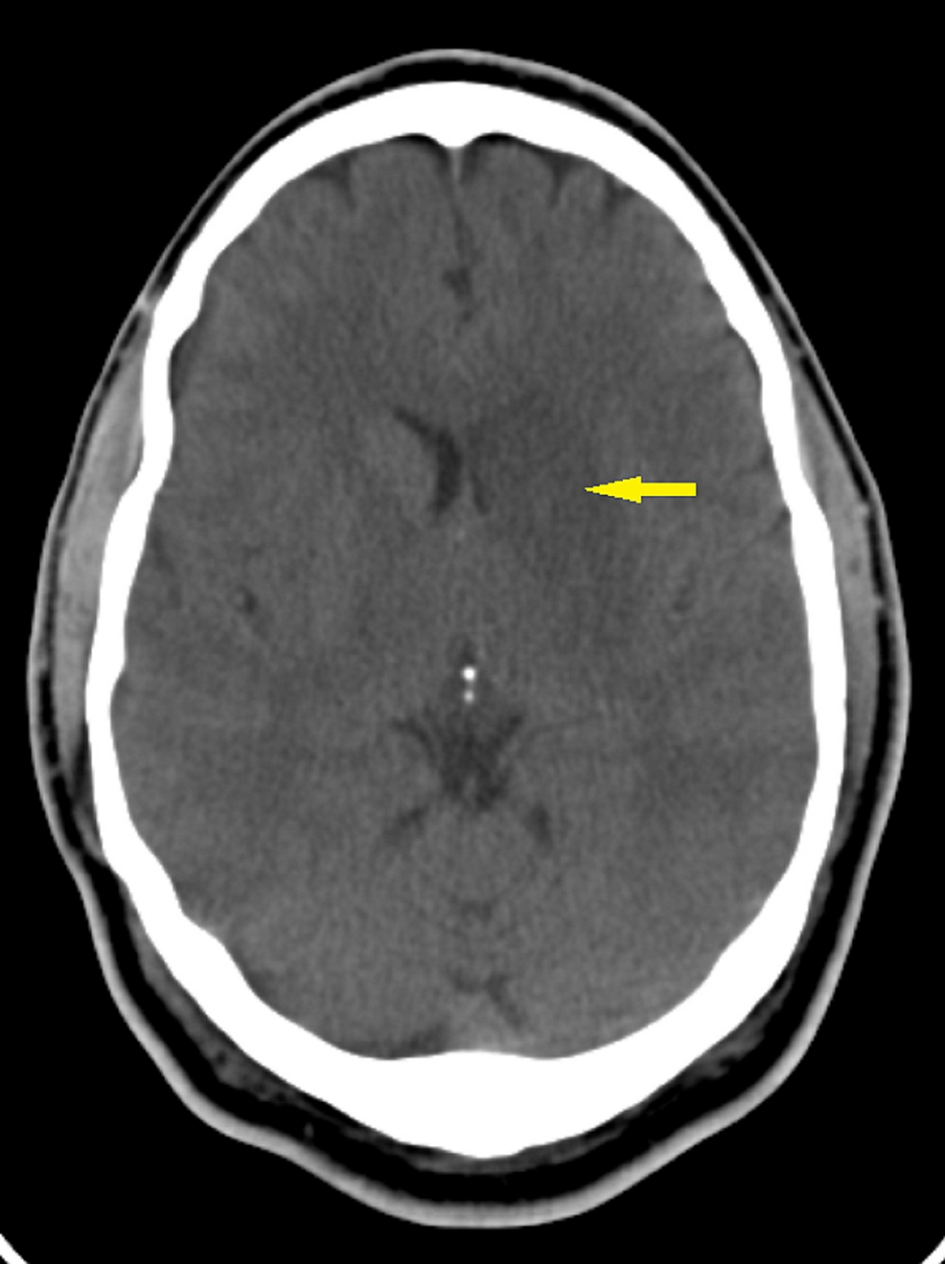
CT of the head showing acute infarct involving the left basal ganglia and head of the left caudate (12/2018)

Mr. G is a 48-year-old African American man who initially presented to the hospital in December 2018 with right-sided weakness, slurring of speech, change in mentation, lethargy, and expressive aphasia. CT head showed a left middle cerebral artery infarct involving the left basal ganglia and head of the left caudate (Figure 1).

Electrocardiogram (EKG) and Holter monitor showed normal sinus rhythm with no atrial or ventricular tachyarrhythmias. Transesophageal echocardiogram (TEE) showed Lambl's excrescences of the aortic valve with no patent foramen ovale, no right to left intracardiac shunt, and no aortic atheroma. Before this event, Mr. G had no past medical history or psychiatric history. He reported using cannabis since age 12, smoking half a pack of cigarettes since age 12, and consuming one to two drinks of alcohol once in two weeks. He subsequently underwent mechanical thrombectomy. After 23 days of hospital stay, Mr. G was discharged to the rehabilitation center. On discharge, Mr. G had mixed non-fluent aphasia and right hemiplegia. He was on aspirin 81 mg, clopidogrel 75 mg, metoprolol 25 mg daily, and atorvastatin 40 mg at bedtime. The patient regained slight power in the right leg and ambulated with an assistive device with rehabilitation therapy. He was sent home after three months in April 2019. However, Mr. G continued smoking a pack of cigarettes a day and was non-compliant with medications.

**Figure 2:**
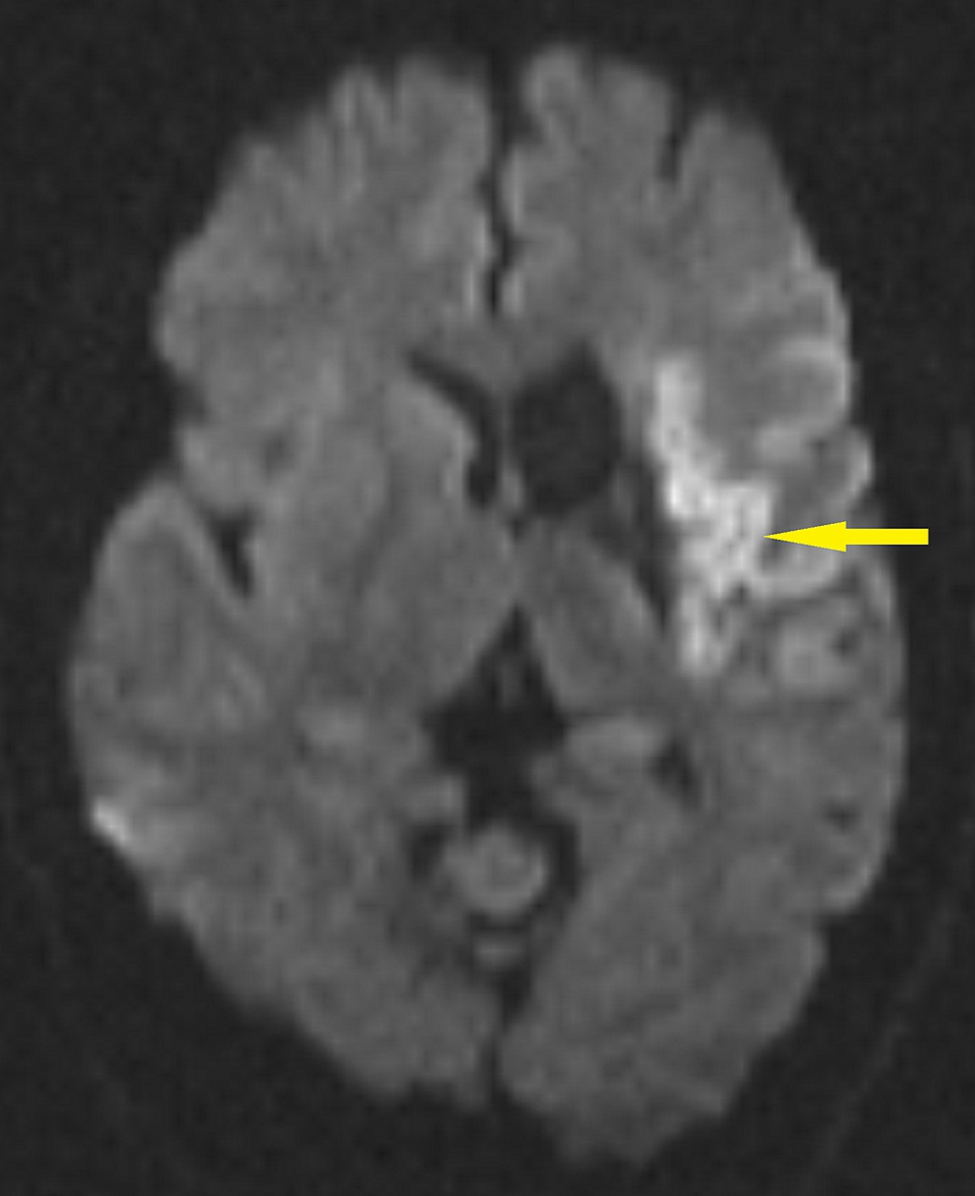
MRI brain axial DWI showing large acute infarct involving the left frontal lobe and left insular cortex (07/2019) DWI, Diffusion Weighted Imaging

Mr. G presented to the hospital again in July 2019 with worsening of aphasia, confusion, and change in behavior for one week before this hospital admission. He had expressive aphasia, so history was unable to be obtained. Computerized tomography of the head (CT head) and Magnetic Resonance Imaging (MRI) brain showed an acute infarct in the left frontal lobe and encephalomalacia in the left basal ganglia/caudate nucleus, consistent with prior infarct in December 2018 (Figure 2).

Electrocardiogram (EKG) and Holter monitor showed normal sinus rhythm with no atrial or ventricular tachyarrhythmias. Furthermore, he was out of the time window for administering the Tissue plasminogen activator (tPA). Mr. G exhibited inappropriate laughter during the second day of hospital admission, and pseudobulbar affect (PBA) was considered. He was started on donepezil for possible vascular dementia; however, this was later discontinued. He was then discharged home on aspirin 81 mg daily, clopidogrel 75 mg daily, atorvastatin 40 mg at bedtime, and nicotine patch 21 mg/24 hours for smoking cessation. He also mentioned that he was advised to take escitalopram 10 mg daily for his mood. However, there was no psychiatric diagnosis for starting escitalopram. Nevertheless, he was again non-compliant with medications.

After four weeks of hospital discharge, Mr. G was brought to the emergency department in August 2019 with confusion, disorganized behavior with talking to self, and frequent episodes of agitation. On one instance before this visit, according to his partner, he was wandering in his area and appeared lost and was confused about his location. His partner was concerned about Mr.G's self-destructive behavior by increased use of marijuana and medication non-compliance. He denied hallucinations, anxiety, depression, or manic symptoms. He was diagnosed with adjustment disorder and sent home with a recommendation to start fluoxetine 20 mg daily. Mr. G, however, did not want to start taking it. 

He was brought to the emergency department again in October 2019 with an irritable mood and allegedly punching his partner. Mr. G had severe expressive aphasia, so his partner primarily provided the history. According to her, the patient's personality changed after his two strokes. He was more irritable and smoking more cannabis, and whenever she would confront him, he would allegedly punch his partner. He was diagnosed with possible vascular dementia with behavioral disturbance or substance-induced mood disorder. He again denied hallucinations, anxiety, depression, or manic symptoms. Divalproex 250 mg daily was recommended, and he was released from the emergency department. His partner, however, did not want Mr. G to return to her apartment due to safety concerns, and he was taken into police custody and released after a few hours.

Mr. G presented to the neurology outpatient clinic in December 2019, and PBA was again considered with inappropriate laughter. He was non-compliant with aspirin, clopidogrel, and atorvastatin. He was advised to be compliant with medications and continue with rehabilitation therapy.

Mr. G was brought to the emergency department multiple times between June 2020 to August 2020 with aggressive behaviors, punching his partner, and medication non-compliance. He was recommended to be compliant with medications and advised to follow up with neurology; however, Mr. G did not follow up as instructed. During one of the visits to the emergency department, he was prescribed divalproex 250 mg twice daily with a diagnosis of a major neurocognitive disorder, possibly due to vascular disease, with behavioral disturbance. He was told to follow up in the psychiatry outpatient clinic; however, he did not follow up. During another emergency department visit, he was diagnosed with a major depressive disorder without psychotic features, in partial remission, and escitalopram 10 mg daily was prescribed. Again, there was difficulty obtaining a history from the patient due to expressive aphasia. 

**Figure 3:**
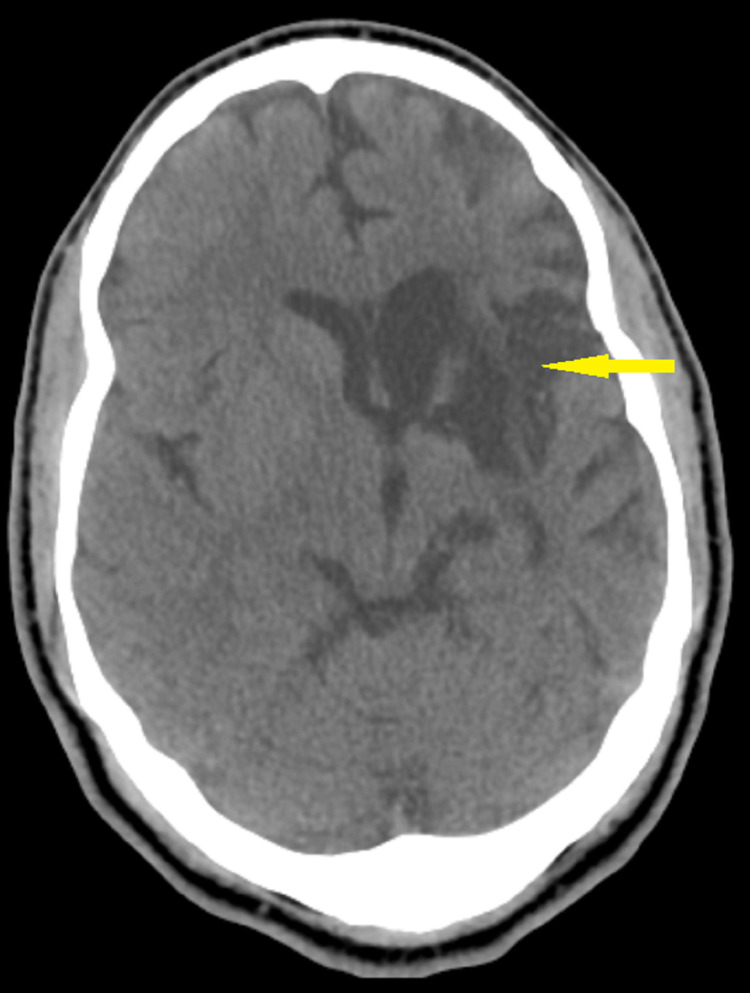
CT of the head showing chronic infarction of the left basal ganglia/corona radiata and the insula and frontal opercular region (6/2021)

In June 2021, his partner brought Mr. G to the hospital with complaints of not taking any medications, not eating, and being physically and verbally abusive. She reported that Mr. G would smoke cannabis multiple times a day, and he would hit his wife when she would not give money for cannabis. She also noted him internally preoccupied, laughing and crying to himself. These episodes of uncontrollable laughter were stereotypical and paroxysmal, incongruent with mood. He was also reported to have inappropriate smiling and laughter during this admission. CT head showed chronic infarction of the left basal ganglia/corona radiata and the insula and frontal opercular region, consistent with prior strokes. No acute hemorrhages, no acute infarction, or mass effects were noted (Figure 3).

EKG showed normal sinus rhythm. He was diagnosed with major depressive disorder, multiple episodes, without psychotic features, in partial remission, and cannabis use disorder. During this admission, divalproex 500 mg twice daily for mood stabilization, escitalopram 10 mg daily, and later increased to 20 mg daily for depression were given. Also, nicotine 21 mg patch for tobacco use disorder and dextromethorphan-quinidine 20-10 mg twice daily for pseudobulbar affect were started. In addition, he was also started on aspirin 81 mg, clopidogrel 81 mg daily, atorvastatin 10 mg at bedtime, and metoprolol 50 mg daily. After 24 days of hospital stay, he was discharged home on these medications in July 2021.

Mr. G had a follow-up psychiatry clinic visit in September 2021. He reported improvement in mood and aggressive behavior with a resolution of uncontrollable laughing spells with a combination of escitalopram 20 mg daily, divalproex 500 mg twice daily, and dextromethorphan/quinidine (20/10 mg) twice daily. 

## Discussion

PBA is a condition characterized by emotional lability with frequent uncontrollable outbursts of laughing or crying. Charles Darwin initially described involuntary emotional expressions of laughing and crying in his book in 1872, “The Expression of the Emotions in Man and Animals” [7]. 

PBA can present a challenge to clinicians, and it is often underdiagnosed or misdiagnosed. Clinicians can find it difficult to distinguish this from mood disorders or to diagnose this disorder in the context of underlying mood disorders. In addition, the delay in the diagnosis can significantly impact patients' quality of life.

In 1969, Poeck summarized PBA symptoms based on four criteria (Table 1). Poeck discussed that emotional stimuli could precipitate episodes of emotional expression; however, he did not discuss that the symptoms can be out of proportion to the stimulus [8].

**Table 1:** Summary of Poeck Criteria for PBA

Poeck Criteria	Description
Criteria 1	Episodes are inappropriate to the situation and can be precipitated by nonspecific stimuli.
Criteria 2	There is no close relation between emotional expression and the patient’s mood.
Criteria 3	The episodes are relatively stereotyped, paroxysmal, and ritualistic.
Criteria 4	There are no episodic mood changes that appropriately correspond to the episodes.

Our patient described in this case had all these four criteria with paroxysmal, stereotypical episodes inappropriate to the situation with no close relation with his mood and no episodic mood changes. 

In 2006, Cummings et al. described comprehensive PBA criteria mentioning that symptoms of emotional expression can be out of proportion to any provoking stimulus and that the symptoms are incongruent to the corresponding emotional state (Table 2) [9].

**Table 2:** Summary of Cummings Criteria for PBA

Cummings Criteria	Description
Criteria 1	Episodes of emotional expression represent a change from the person’s usual emotional reactivity.
Criteria 2	Emotional reactivity is inconsistent with the person’s mood or the corresponding mood state.
Criteria 3	The emotional response is more than any provoking stimulus.
Criteria 4	Repetitive episodic emotional disturbances cause significant distress or social or occupational impairment.
Criteria 5	Sudden emotional occurrences of expression are not accounted for by another psychiatric or neurological disorder.
Criteria 6	The symptoms are not a result of the direct effect of a drug or medication use.

Mr. G's emotions represented a change from his usual emotional reactivity since he was a high-functioning individual with none of these emotions before the stroke. The episodes of uncontrollable laughter were inconsistent with his mood. Emotional expressions and aggressive behaviors were out of proportion to any provoking stimulus. There was significant social impairment, and the partner was unwilling to stay with the patient. There is no history of traumatic brain injury. Major Neurocognitive Disorder, possibly due to vascular disease, with behavioral disturbance, was also considered with a history of recurrent strokes. The Mini-Mental State Examination (MMSE), Montreal Cognitive Assessment (MoCA), and neuropsychological testing are commonly utilized to screen patients for neurocognitive disorders. The combined sensitivity for detecting dementia using the MMSE, MoCA, and other screening tests is 81 percent, and the specificity is 89 percent [10]. However, these tools were not utilized in our patient described in this case. In addition, Cannabis use disorder was also considered in the differential diagnosis. Evidence for long-term neurocognitive impairment associated with cannabis use is mixed. Cognitive impairment appears to depend on the dose of cannabis, which resolves with abstinence, usually within a few days to a month [11]. Mr. G has a long history of cannabis use with increased use since the stroke; however, the history of paroxysmal episodes of uncontrollable laughter and crying incongruent with his mood is most likely related to PBA. 

PBA is associated with various neurological conditions, including stroke. PBA is caused by disturbances in the neurotransmitters and emotional centers involved in inhibiting emotional expression [12]. However, the exact neurological pathways involved in PBA are not fully understood.

Recognizing PBA is essential, and the Center for Neurologic Study- Lability Scale (CNS-LS) is a validated clinical tool used to measure self-reported symptoms of PBA [13]. The CNS-LS includes seven items with four items related to laughter and three for crying, with each item scored on a 5-point scale for a total score ranging from seven (no symptoms) to 35 (maximum). A CNS-LS score of ≥ 13 indicates the presence of PBA symptoms, whereas a score < 13 denotes an absence of PBA symptoms. 

The Pathological Laughing and Crying Scale (PLACS) is another tool validated for evaluating PBA symptoms in stroke [14]. It comprises 18 questions with two items for the presence of sudden episodes of crying or laughing, eight items for laughing, and eight for crying. Each item scored from zero (normal) to three (excessive emotional lability), and a score of 13 or more indicates PBA.

Mr. G had his first stroke in December 2018 with expressive aphasia. PBA was considered when he exhibited inappropriate laughter after his second stroke in July 2019. PBA was again considered in December 2019 when Mr. G was noted to have inappropriate laughter during a neurology outpatient clinic visit. However, no further screening was performed to diagnose PBA, and no medication was started for this during that visit. He was subsequently lost to follow-up with neurology. There was difficulty obtaining a history from the patient due to expressive aphasia. Mood disorder was also considered during one of the emergency room visits with recommendations of escitalopram and divalproate. Nevertheless, the patient was non-compliant with medications.

Regarding PBA treatment, clinicians had tried various classes of off-label medications till 2010. These medications target serotonin, glutamate, or dopamine receptors [15]. Selective Serotonin Reuptake Inhibitors (SSRIs) were considered as a first-line treatment option, and Tricyclic Antidepressants (TCAs), Serotonin-Norepinephrine Reuptake Inhibitors (SNRIs) noradrenergic reuptake inhibitors, other antidepressants (Mirtazapine), dopaminergic agents, and uncompetitive N-methyl-D-aspartase (NMDA) receptor antagonists were tried as second-line agents. In October 2010, FDA approved the drug dextromethorphan (20 mg)/quinidine (10 mg) for the treatment of PBA [16]. The exact mechanism of action is not known. Dextromethorphan may relieve PBA symptoms by acting as an SNRI inhibitor, NMDA receptor antagonist, sigma-one receptor agonist, and nicotinic receptor antagonist [17]. Quinidine increases the serum concentration of dextromethorphan by blocking its rapid metabolism. 

In this patient, dextromethorphan/quinidine was started two years and eight months after his first stroke. During two month follow-up, he reported improvement in mood and aggressive behavior with a resolution of uncontrollable laughing spells with a combination of escitalopram 20 mg daily, divalproex 500 mg twice daily, and dextromethorphan/quinidine (20/10 mg) twice daily. In addition, he reported compliance with medications without any side effects. 

An interesting fact was that this patient had aggressive behavior in addition to uncontrollable laughing spells. In addition, he was compliant with his medications after starting dextromethorphan/quinidine. 

## Conclusions

We present a case of PBA with aggressive behavior in addition to uncontrollable laughing spells. PBA should be strongly considered in patients with stroke, and early recognition and management may help patients and their families. In addition, major neurocognitive disorders, substance use disorders, and other psychiatric disorders should also be considered. Clinicians should utilize the CNS-LS and the PLACS tools to screen for PBA, especially in the context of stroke or other neurological disorders. Dextromethorphan/Quinidine may be considered as 1st line treatment for PBA. However, further research may be needed to assess the effect of Dextromethorphan/Quinidine on improving medication compliance in PBA. 
